# Error-related negativity predicts failure in competitive dual-player video games

**DOI:** 10.1371/journal.pone.0212483

**Published:** 2019-02-28

**Authors:** Yusuke Yokota, Takahiro Soshi, Yasushi Naruse

**Affiliations:** Center for Information and Neural Networks (CiNet), National Institute of Information and Communications Technology, and Osaka University, Kobe, Hyogo, Japan; Kochi University of Technology, JAPAN

## Abstract

Along with improvement in electroencephalogram (EEG)-measurement technology, limitations on the situations in which data can be recorded are gradually being overcome. EEG measurement in real environments has become increasingly important as a means to monitor brain activity in our daily lives, such as while playing consumer games in the living room. The present study measured brain EEG activity while two players engaged in a competitive consumer baseball game in conditions that closely resembled daily life. The recorded brain activity was thus likely related to natural mental reactions and cognitive function that occur in similar daily life activities. To measure the EEG from participants who freely moved while playing the game, we developed EEG devices that incorporated a wireless time synchronization system using Global Positioning Satellite (GPS) signals. These devices stamped the time obtained from the GPS signals onto each data sample, which was then used to synchronize the data that were recorded by different devices. When the batter in the game swung and missed, the error-related negativity component of the event-related EEG potential was strongly evoked in frontal electrodes of the participant controlling the batter. Furthermore, the error-related negativity was modulated according to who was winning and by how much. Thus, here we have demonstrated "real-world" brain activity using a competitive consumer game, which increases intrinsic participant motivation.

## Introduction

Many people enjoy computer games all over the world. Traditionally, playing these types of games has required special hardware. Smartphone technology has become dramatically more sophisticated in recent years, allowing people to more easily play games. Players complete the missions and goals prepared inside virtual gaming situations (e.g., beat the enemy, get a high score or gather items). Previous studies have reported that people tend to play games because they are spontaneously interested in and enjoy the games themselves, even without reward money [1–3]. Therefore, computer games are useful as a means to enhance a participant’s intrinsic motivation during an experiment.

In typical experiments that use electroencephalogram (EEG) machines to record brain activity, visual stimuli usually only have simple physical properties. This is because it allows the identification of specific brain activity that changes depending on the limited stimulus property (e.g., stimulus intensity). Therefore, participants rarely find these kinds of experiments with simple stimuli to be fun. Although these kinds of basic experiments are very important in the field of neuroscience for discovering mechanisms that underlie human brain function, they are not suitable for revealing "real-world" brain activity that occurs in our daily lives when we experience fun events.

However, a fun and interactive game is problematic because players are simultaneously exposed to numerous factors related to the events occurring in the game. Therefore, analyzing which information is specific to newly obtained brain activity related to a game event is difficult. This can be overcome if we use brain activity markers for which generative mechanisms have already been reported. Then, we are able to identify the functional information reflected by the brain activity while playing games. Indeed, recent studies have measured brain activity while participants played games [4–6]. These studies used custom-developed games because they could deliver precise timing of game-event onsets to a playing log for EEG data analysis. However, these custom-developed games were very simple. At the same time, while high-quality consumer games are complex and enable players to operate freely, we cannot obtain an accurate data log, and detecting game events with precise timing is a problem. Because of the advantage of realism and the fact that consumer games have the added advantage of enhancing intrinsic participant motivation, we developed a data-recording hardware system that can extract game events from a consumer game with precise timing and measure "real-world" event-related potentials (ERPs) when people play the game in an ordinary life context.

We used a commercial baseball game in which two players compete. In a single-player game setting with the computer as the opponent, players become bored once they arrive at a winning strategy. In a competitive game setting, establishing a specific winning strategy is relatively difficult because the players continuously change their strategies in response to each other. Thus, the desire to defeat a human opponent should enhance a participant’s intrinsic motivation when playing competitive games. The advantage of using the baseball game is that we can control the physical properties of the visual stimuli between batters and pitchers. Because players controlling the batters and the pitchers (referred to as batters and pitchers) observe the same game screen at the same time, the physical properties of the visual stimuli are same for the two players throughout the experiment. Furthermore, because the players pay attention to the positions of the ball and the bat marked by the cursor, the attended areas of the screens can be controlled for each player separately. Under these physical conditions, strikes and balls should evoke different cognitive responses and different brain activity.

The present study focused on the error-related negativity (ERN), an ERP relating to error events [7,8]. The ERN is evoked between 0 and 100 ms after an error occurs, peaking at about 50 ms post-error. The ERN is highly versatile because it is independent of task and age [9–12]. Moreover, motivational and personal factors are reflected in the magnitude of the ERN. Previous study found that larger ERNs were evoked when participants focused on accuracy rather than response speed for their task responses [8]. Punishment resulted in larger ERNs to errors than did reward [13], and larger ERNs have been found in participants who are more absorbed in the task [14]. Regarding the relationship between personal factors and ERN magnitude, larger ERNs have consistently been observed in anxious participants [15]. Additionally, the ERN is sensitive to individual error responses that depend on personal factors such as conscientiousness [16], socialization [17], and self-efficacy [18,19]. The ERN does not occur when participants do not notice their own mistakes.

Therefore, we expected that the ERN amplitudes related to game-event errors would be modulated according to gaming situations such as score difference. In particular, we predicted that batter ERN would be modulated according to the magnitude of the mistakes that the batter noticed or felt when a strike was called. As mentioned above, because the ERN can be modulated by personal factors, we assessed personal factors using self-reporting questionnaires. We used the Barratt Impulsiveness Scale 11 (BIS11[20,21] to quantify impulsivity, and the Behavior Inhibition/Activation System (BIS/BAS) scales [22,23] to quantify the tendency to avoid unpleasant future behavioral consequences and the motivational preference for favorable results. Then, we investigated the relationship between the ERN and these factors. We also focused on P300, a positive potential at approximately 300 ms after stimulus presentation [24]. The P300 reflects various cognitive processes such as the engagement of attention [25–27]. We therefore expected that the amounts of attention on the batting/pitching results should affect P300 amplitude. In summary, we used a competitive consumer game to increase intrinsic participant motivation, and focused on ERN and P300 surrounding game events.

## Materials and methods

### A system for extracting game events from a consumer game

**Fig 1:**
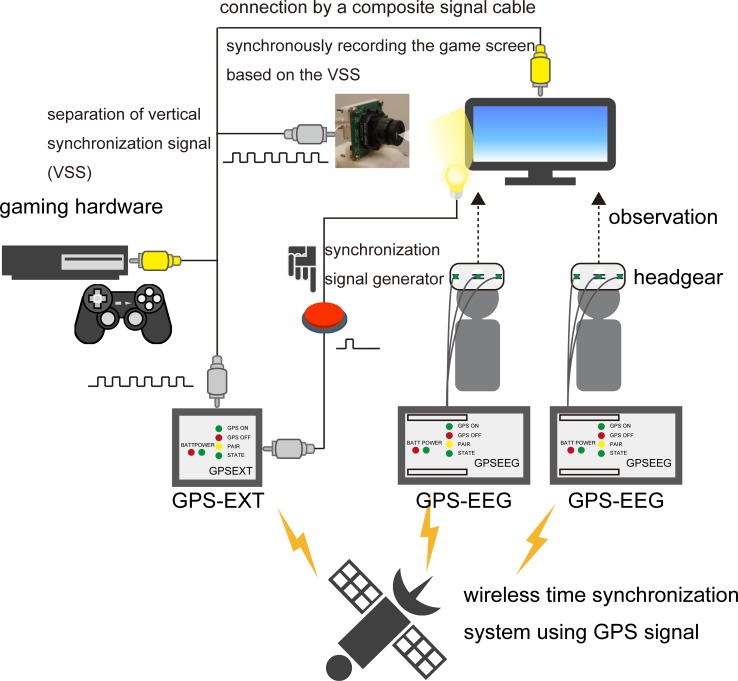
Wireless synchronization system among Electroencephalograms (EEGs), External devices (EXT), and the consumer game using a GPS signal. This system can extract the onset time of game events from a composite video signal.

We developed a wireless data-recording system that can measure the EEG from participants who are freely moving while playing the game and also precisely extract the timing of game events from consumer games. A schematic illustration of the recording system is shown in Fig 1. This system consists of three subsystems.

The first subsystem is a wireless time synchronization system using Global Positioning Satellite (GPS) signals. Typical neuroscience studies use the event-synchronization method in which a trigger signal from a stimulus is input to an external port of an EEG device, allowing stimulus onset to be recorded accurately with millisecond resolution. Additionally, simultaneous EEG measurements from multiple people (as in the present study) should share the trigger signal among separate EEG devices. In other cases, the trigger signals are generally transmitted via a wired cable to the devices. However, wired connections restrain participant movement. For this study, we therefore developed an EEG device and external input device that can receive GPS signals and establish wireless synchronization between EEG data and external input data. The GPS signal contains time information, and the time accuracy has greater than millisecond resolution. Our device can add a time-stamp for each sampled datum, which can then be used to synchronize the EEG and external data that were recorded by different devices. We used a separate EEG device (GPS-EEG) for each player and one external input device (GPS-EXT) for acquiring trigger signals.

The second subsystem is the system that for recording every video frame of the consumer game. The gaming hardware and the monitor were connected by a composite signal cable. The composite video signal includes 60 Hz rise-up vertical synchronization signals according to the screen refresh rate (60 Hz). By synchronizing the video camera for recording the game screens with the vertical synchronization signals, we could record every video frame of the game screens. The composite signal was separated into three branches. The first branch was connected to the monitor. The second branch was connected to the video camera for recording the game screen. We used a video camera (MCM-4304; Micro Vision Co., Ltd., Niigata, Japan) that could synchronously record the video based on the vertical synchronization signal extracted from the composite signals. The recorded video was monochrome with a resolution of 320 × 240 pixels. The vertical synchronization signal that was extracted from the third output branch was sent to the GPS-EXT device.

**Fig 2:**
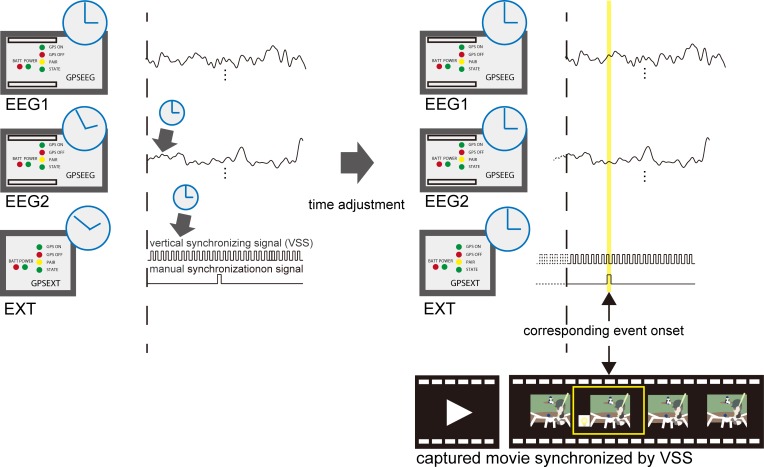
A schematic illustration of time adjustment. All EEG and EXT signals are time-stamped with information obtained from the GPS signal. We adjusted the time information between EEG data and the recorded movies using the vertical synchronization signal (VSS) signal that was extracted from the composite signal.

The third subsystem is a synchronization signal generator. Using the two subsystems mentioned above, the vertical synchronization signal and EEG can be synchronized via the time-stamp based on the GPS signal. However, this information cannot be used to judge which video frames recorded by the camera correspond to which vertical synchronization signals recorded by GPS-EXT device. To solve this problem, we added a synchronization signal to the video frame and GPS-EXT device at the start and end of the game. The synchronization signal was generated by manually pushing a button. When the button was pushed, the device transferred the synchronization signal to the GPS-EXT device, and an LED light was simultaneously turned on and the light was recorded by the camera. These three system components enabled us to synchronize all game events with the EEG data obtained while playing the game. A schematic illustration of the time synchronization system is shown in Fig 2.

### Experimental devices

EEG data were measured by a wearable data-recording system comprising a GPS-EEG device that could be attached to custom-designed headgear (Sawamura Prosthetics and Orthotics Service, unique development, JPN). The GPS-EEG device had 8-channels with active dry electrodes for EEG recording and Bluetooth signal transportation (Miyuki Giken, original development based on Polymate Mini AP108 [W52-D50-H20 mm, 80 g], JPN). The headgear allowed dry EEG electrodes (Unique Medical, unique development, Japan) [28] at the fronto-central (FC3, FCz, FC4) and occipital sites (O1, O2). To measure electrooculograms (EOGs), two electrodes were placed on the forehead and right temple of each participant. All recorded signals were referenced to the left mastoid and the ground electrode was placed on the right mastoid. Data were sampled at 500 Hz.

We used the PlayStation 3 (Sony, CECHH model) and Jikkyō Powerful Pro Yakyū 2013 (Konami Digital Entertainment Co., Ltd., JPN) as the consumer hardware and software, respectively. This game software is a popular Japanese baseball video game. Participants sat on a couch and observed the monitor located 170 cm in front of them.

### Participants

We recruited the participants from the public. As a result, nineteen participants (17 male, 2 female; age range: 21–40 years) took part in this study. However, since there were so few female participants, their data were excluded from the present study’s analysis. All participants had normal hearing and normal or corrected-to-normal vision. Participants had never played the baseball game that we used in this study. Participant pairs who played the game competitively were acquaintances. Participants provided informed written consent after the details of the procedure had been explained and before the experiment. All experimental procedures were approved by the Ethical Committee for Human and Animal Research of the National Institute of Information and Communications Technology. All experiments were performed in accordance with the ethical standards described in the Declaration of Helsinki.

### Experimental procedure

All participants practiced before the EEG experiment until they learned how to operate the game. Nippon Professional Baseball has two professional baseball leagues (Central and Pacific Leagues). In this experiment, the participants used the teams comprising the all-star representatives from each league. The participants played three nine-inning games in the EEG experiment to increase the number of game-event trials, without any restrictions on physical movement.

### Psychological measures

To assess intrinsic motivation for playing the game, we administered a self-reporting questionnaire. Because intrinsic motivation must be evoked and intention has to be directed towards completing goals, these behaviors are quite different from the impulsivity often observed in negative behaviors, such as non-attention, non-planning, and non-controlling behaviors. Therefore, we also recorded impulsivity traits using the Barratt Impulsiveness Scale 11 (BIS11[20,21] and Behavior Inhibition/Activation System (BIS/BAS) scales [22,23].

Behavioral impulsivity was evaluated with the BIS-11. The BIS-11 focuses on three impulsivity traits: attentional (AI: 5, 6, 9, 11, 20, 24, 26, 28; 8–32 scores), motor (MI: 2–4, 16, 17, 19, 21–23, 25, 30; 11–44 scores), and non-planning (NPI: 1, 7, 8, 10, 12–15, 18, 27, 29; 10–40 scores). Participants responded to each question using a four-point Likert-like scale (4 = very true for me; 3 = somewhat true for me; 2 = somewhat false for me; 1 = very false for me).

Behavioral properties related to inhibition and activation regulate the avoidance of unpleasant future behavioral consequences and the motivational preference for favorable results, respectively, and were assessed with the BIS/BAS scales [22,23]. The BIS component (No. 2, 8, 13, 16, 19, 22, 24; 7–28 scores) evaluates avoidance of unpleasant future behavioral consequences. The BAS assesses motivational preference for favorable results, consisting of drive (D: 3, 9, 12, 21; 4–16 scores), reward responsiveness (RR: 4, 7, 14, 18, 23; 5–20 scores), and fun seeking (FS: 5, 10, 15, 20; 4–16 scores). The participants scored each item with a four-point Likert-like scale (4 = very true for me; 3 = somewhat true for me; 2 = somewhat false for me; 1 = very false for me).

After the end of the game, participants filled out the Game Experience Questionnaire (GEQ) [29,30], which is a self-evaluation of game experience. The GEQ consists of the following three modules: ‘Core’ (33 items), ‘Social Presence’ (20 items), and ‘post-game' (20 items). The Core module assesses game experience with scores on seven components: ‘immersion’ (6 items; e.g., “It was aesthetically pleasing”), ‘flow’ (5 items; e.g., “I forgot everything around me”), ‘competence’ (5 items; e.g., “I felt skilful”), ‘positive affect’ (5 items; e.g., “I felt happy”), ‘negative affect’ (4 items; e.g., “It gave me a bad mood”), ‘tension’ (3 items; e.g., “I felt annoyed”), and ‘challenge’ (5 items; e.g., “I thought it was hard”). The Social Presence module assesses psychological and behavioral involvement of the player with other social entities using scores on three components: ‘empathy’ (6 items; e.g., “I empathized with the other(s)”), ‘negative feelings’ (5 items; e.g., “I felt jealous about the other(s)”), and ‘behavioral involvement’ (6 items; e.g., “My actions depended on the other(s) actions”). The post-game module uses scores on four components to assess how players felt after they had stopped playing: ‘positive experience’ (6 items; e.g., “I felt like a victory”), ‘negative experience’ (6 items; e.g., “I felt bad”), ‘tiredness’ (2 items; e.g., “I felt exhausted”), and ‘returning to reality’ (3 items; e.g., “I found it hard to get back to reality”). Participants scored each item with a five-point Likert scale (4 = extremely; 3 = fairly; 2 = moderately; 1 = slightly, 0 = not at all).

### EEG data analyses

EEG analyses were performed using MATLAB (MathWorks, Inc., Natick, MA, USA). A digital finite impulse response bandpass filter (1–20 Hz, order 500) was applied to the continuous EEG data. Subsequently, we used the informax ICA installed in the EEGLAB toolbox [31] to perform an independent component analysis that removed eye-movement artifacts. We then extracted the EEG data and movie frames between the start and end of each game based on the synchronization signal (Fig 2).

**Fig 3:**
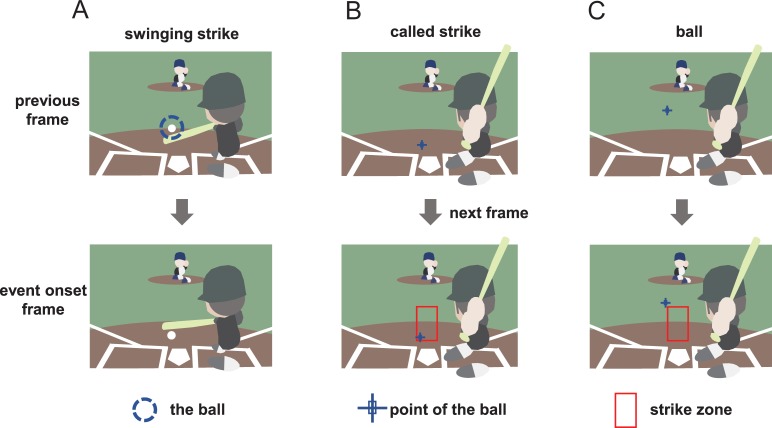
A schematic illustration of the onset criteria for the three ball/strike events. We visually identified the onset of game events from the recorded movies.

A schematic illustration of the onset criteria for game events is shown in Fig 3. We visually identified the onset of ball event and two kinds of strike events. A swinging strike is an event in which the batter swung the bat, but the batter did not hit a ball thrown by pitcher. A called strike is another type of strike event in which the pitcher threw the ball into strike zone, but the batter did not swing the bat. We did not analyze other events such as hits, fouls, or errors. For swinging strikes (Fig 3A), on the basis of the previous frame (Fig 3A upper), we cannot judge whether the bat hits the ball. However, from the next frame (Fig 3A lower), we can judge this event as a swinging strike. Therefore, we selected the frame (Fig 3A lower) in which the batter had swung and missed the ball as the event onset. For called strikes and balls (Fig 3B and 3C), on the basis of the previous frame (Fig 3B and 3C upper), we cannot judge whether the ball has entered the strike zone. However, from the next frame (Fig 3B and 3C lower), we can judge this event as a called strike or ball, as the strike zone appears in this frame. Therefore, we used this frame (Fig 3B and 3C lower) as the event onset.

For each EEG channel, the EEG data were divided into 1000 ms epochs (−400 to +600 ms) based on the ball/strike event onsets. Epochs containing data with maximum absolute amplitudes greater than 70 μV at any of the channels (FC3, FCz, FC4, O1, or O2) were excluded from subsequent analyses. Baseline correction was performed using the averaged amplitude from −600 to −400 ms. In this study, five of the seventeen participants played games against two opponents. For these five participants, we only analyzed the data collected from their first experiment.

### Statistical analyses

We used R version 3.5.1 [32] for statistical analysis of EEG data. For ERP amplitude, we employed a linear mixed model (LMM) using the lme4 package and the lmerTest package for statistical analysis. We defined a Game Event (ball, swinging strike, or called strike) and Player Position (batter or pitcher) as fixed effects. We defined a Participant and the Participant’s Age as random effects. Our LMM model was: EEG components ~ Game Event * Player Position + (1 | Participants) + (1 | Participant’s Age). For the calculated LMM model, we used analysis of variance (ANOVA) using the anova function of the stats package in R. For post-hoc analysis, we calculated pairwise differences of the least-square means for factor pairs of interest using the difflsmeans function. The p values were corrected for multiple comparisons using the Bonferroni method. We used the average ERP values obtained at FC3, FCz, and FC4 for statistical analysis, because these are the locations at which error-related ERPs have most commonly been observed.

We used MATLAB 2015b for statistical analysis of self-evaluation questionnaires. Participants filled out the BIS11 and BIS/BAS self-evaluation questionnaires related to their personality (behavioral inhibition and activation) and the GEQ, which was related to their subjective experience of the games. To determine the relationship between these evaluations and brain activity, we calculated the correlation coefficient between the questionnaire scores and the ERP amplitudes. For the BIS11, we used 2nd order factors (attentional, motor, and non-planning) for calculating the correlation. For the GEQ, we used each GEQ item to calculate the correlation with brain activity. Data analysis of the GEQ was conducted for 13 participants. Because five participants did not complete the GEQ questionnaire after the experiment, data analysis of the GEQ was conducted for 12 participants.

## Results

**Fig 4:**
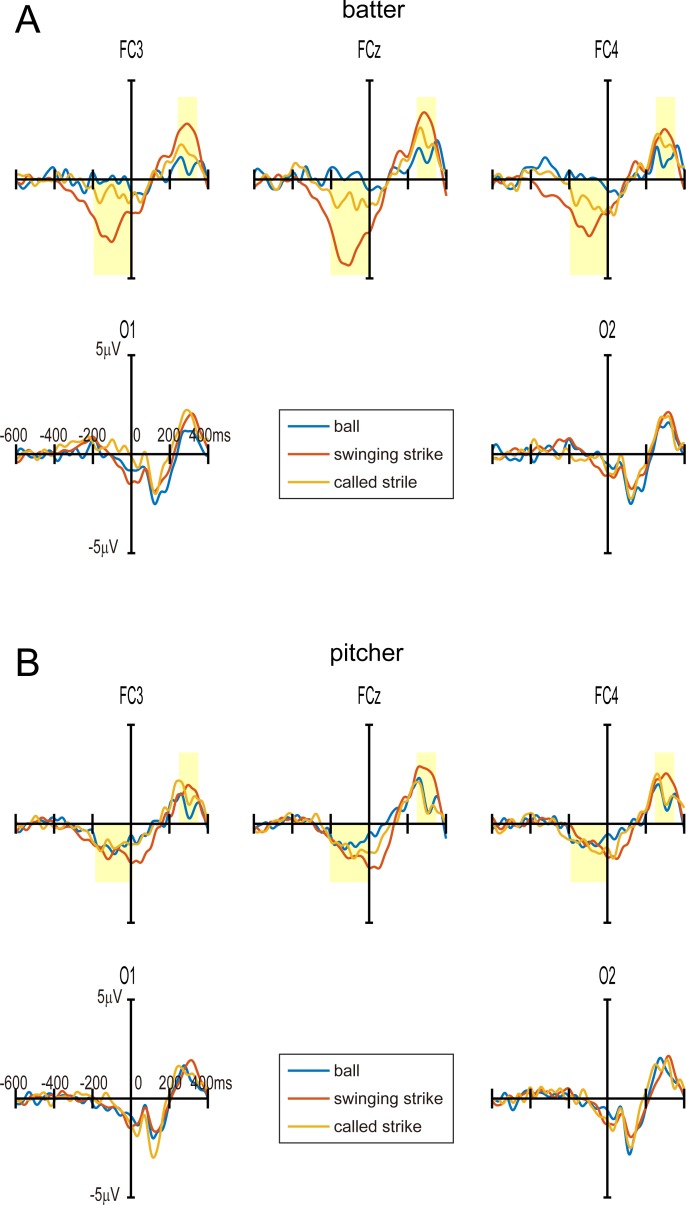
Grand-averaged ERPs for each event (ball, swinging strike, and called strike) for batters and pitchers at the five electrodes. The yellow rectangles indicate the time window for statistical analysis. (A) Grand-averaged ERPs for batters. ERN can be seen before the onset of swinging strikes. P300 can be seen after the onset of all events. (B) Grand-averaged ERPs for pitchers. P300 can also be seen after the onset of all events.

**Fig 5:**
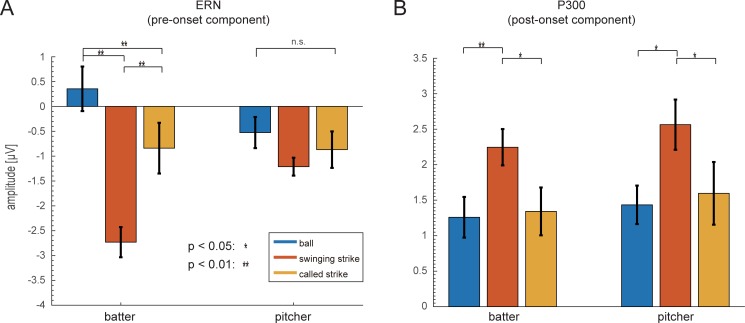
Statistical results for ERN and P300 analysis. (A) Statistical results for ERN. Error bars indicate the standard error. We observed significant differences in ERN amplitude of batters between each event type. (B) Statistical results for P300. Error bars indicate the standard error. We observed significant differences in the P300 amplitudes of batters and pitchers between swinging strikes and balls.

In this study, epochs containing data with maximum absolute amplitudes greater than 70 μV in any of the channels (FC3, FCz, FC4, O1, or O2) were excluded. The numbers of rejected epochs (ball, swinging strike, and called strike) and the percentages of rejected epochs are shown for all participants in Table 1. The maximum percentage of rejected epochs was 20%. However, 11 participants had no rejected trials. The average rejected percentage across all participants was less than 3%. The grand-averaged ERPs for each event (ball, swinging strike, and called strike) for batters and pitchers at the five electrodes are shown in Fig 4 and S1 Table. Fig 4A shows the results when batting, and Fig 4B shows the results when pitching. We confirmed a large negative potential before event onset (−200 to 0 ms) and a large positive potential after event onset (+250 to +350 ms) for swinging strikes. Thus, we defined these two ERP components as pre- and post-onset components. The grand-averaged negative and positive potentials of pre- and post-onset components are shown in Fig 5 for the batting and pitching conditions. The results represent the average values at FC3, FCz, and FC4. Error bars indicate the standard error.

**Table 1:** Numbers of rejected epochs (ball, swinging strike, and called strike) and percentages of rejected epochs for all participants.

Position	Batter			Pitcher		
Event	ball	Swinging strike	Called strike	ball	Swinging strike	Called strike
No.1	0 (0%)	0 (0%)	0 (0%)	0 (0%)	0 (0%)	0 (0%)
No.2	0 (0%)	1 (1.03%)	0 (0%)	0 (0%)	0 (0%)	0 (0%)
No.3	0 (0%)	1 (1.19%)	2 (16.7%)	0 (0%)	1 (1.35%)	0 (0%)
No.4	0 (0%)	0 (0%)	0 (0%)	0 (0%)	0 (0%)	0 (0%)
No.5	0 (0%)	0 (0%)	1 (20%)	0 (0%)	0 (0%)	0 (0%)
No.6	0 (0%)	1 (3.12%)	0 (0%)	0 (0%)	0 (0%)	0 (0%)
No.7	0 (0%)	1 (3.70%)	0 (0%)	0 (0%)	0 (0%)	0 (0%)
No.8	0 (0%)	1 (1.30%)	1 (4.17%)	2 (2.90%)	0 (0%)	3 (6.38%)
No.9	1 (2.33%)	0 (0%)	0 (0%)	0 (0%)	0 (0%)	0 (0%)
No.10	0 (0%)	0 (0%)	1 (4.00%)	0 (0%)	0 (0%)	0 (0%)
No.11	0 (0%)	0 (0%)	0 (0%)	0 (0%)	0 (0%)	0 (0%)
No.12	0 (0%)	0 (0%)	0 (0%)	0 (0%)	0 (0%)	0 (0%)
No.13	0 (0%)	0 (0%)	0 (0%)	0 (0%)	0 (0%)	0 (0%)
No.14	0 (0%)	0 (0%)	0 (0%)	0 (0%)	0 (0%)	0 (0%)
No.15	0 (0%)	0 (0%)	0 (0%)	0 (0%)	0 (0%)	2 (3.92%)
No.16	4 (11.4%)	9 (13.8%)	0 (0%)	3 (7.70%)	0 (0%)	4 (12.5%)
No.17	1 (2.17%)	3 (6.52%)	0 (0%)	3 (4.48%)	2 (8.00%)	1 (3.70%)
Averaged rejected percentage	0.937%	1.80%	2.63%	0.886%	0.550%	1.56%

For the pre-onset components, an ANOVA revealed a main effect of Game Event (*F*_2, 16_ = 16.9, *p* < 0.001, η^2^ = 0.19) and a significant interaction between Game Event and Player Position (*F*_2, 16_ = 8.96, *p* < 0.001, η^2^ = 0.10). Subsequent post-hoc tests revealed significant differences in ERP amplitudes for different conditions, such that swinging strikes elicited a larger negative potential than balls or called strikes in batters (t = 7.04, *p* < 0.001; t = 4.32, *p* < 0.01), and called strikes elicited a larger negative potential than balls in batters (t = 2.73, *p* < 0.05).

For the post-onset component, an ANOVA revealed a main effect of Game Event (*F*_2, 16_ = 11.3, *p* < 0.01, η^2^ = 0.11) but no significant interaction. Subsequent post-hoc tests also revealed significant differences in ERP amplitudes for different conditions, such that swinging strikes elicited a higher amplitude than balls or called strikes in batters (t = 3.35, *p* < 0.01; t = 2.78, *p* < 0.05) and swinging strikes elicited a higher amplitude than balls or called strikes in pitchers (t = 2.83, p < 0.05; t = 2.59, p < 0.05).

The pre-onset component was most strongly evoked in batters just before the completion of a swinging strike. Because batters and pitchers saw the same screen, the difference in brain activity associated with the pre-onset component can be attributed to error processing by the batter rather than any difference in visual stimuli. Thus, we identified this negative potential in the batting condition as the ERN.

The post-onset component was most strongly evoked for swinging strikes, and we did not observe any differences for batters or pitchers, or any interaction. This result suggests that the post-onset component is unlikely to reflect error processing. Because the post-onset component was a positive potential around 300 ms after event onset, we identified this potential as the P300.

**Fig 6:**
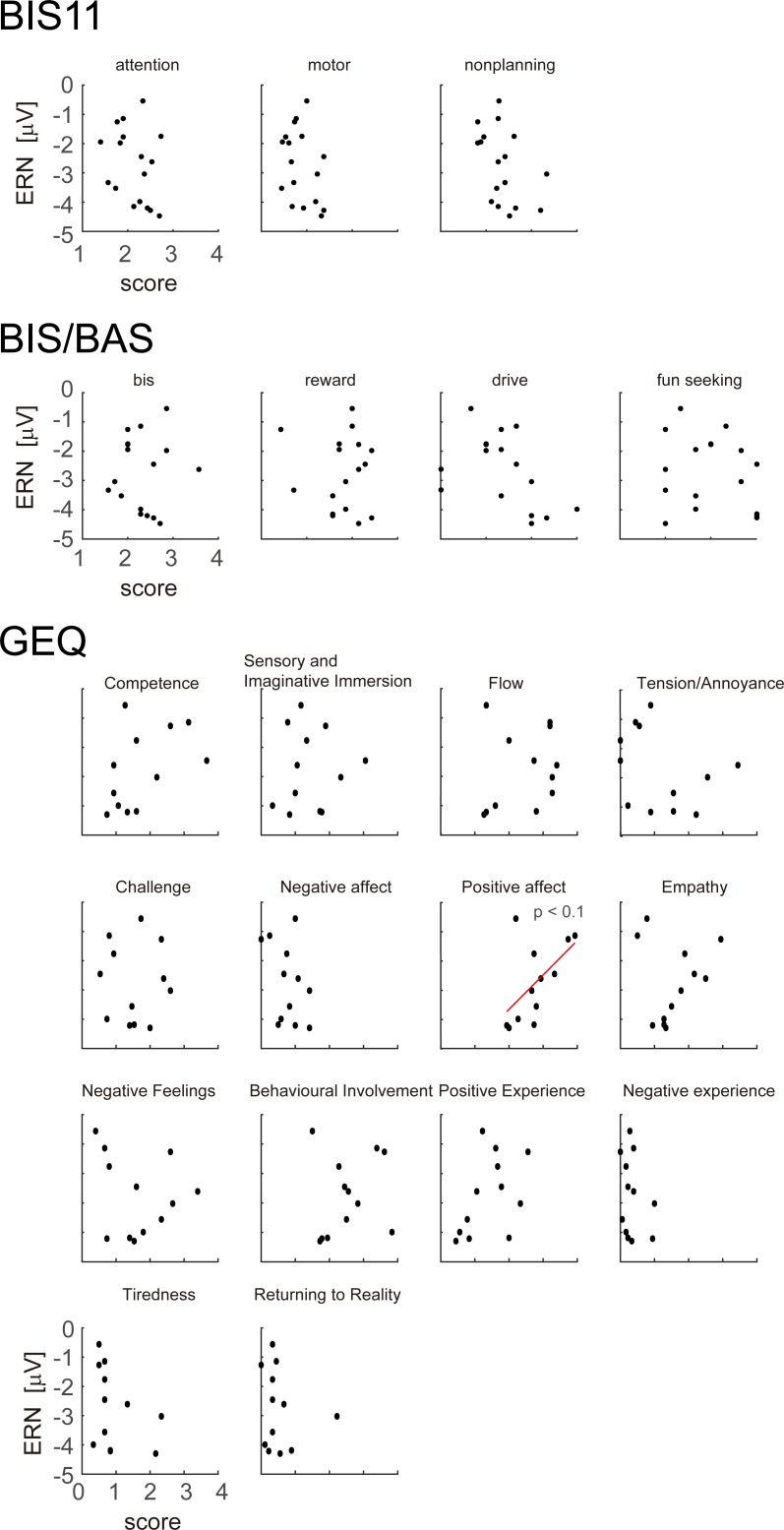
Correlation of ERN amplitude before swinging strikes with scores from the self-evaluation questionnaires (BIS11, BIS/BAS, and GEQ). We found a trend for ERN amplitude to be smaller for higher values of ‘positive affect’.

The mean value, standard deviation, and Cronbach’s alpha for each sub-scale of the self-report questionnaires are shown in Table 2. We investigated whether the magnitude of the ERN correlated with scores on the self-evaluation personality and game questionnaires. The results are shown in Fig 6, Table 2, and S2 Table. None of the BIS11 or BIS/BAS scores were significantly correlated with ERN amplitude. Although there was no significant correlation between GEQ scores and ERN, we found a trend for ERN amplitude to be smaller for higher values of ‘positive affect’ (*p* = 0.0558, *r* = 0.565).

**Table 2:** Mean value, standard deviation, and Cronbach’s alpha for each subscale of the self-report questionnaire and correlational data between ERN amplitude and each score.

Variable			Cronbach’s	Correlation of ERN amplitude	
	M	SD	alpha	*p*	*r*
BIS 11					
attention	2.142	0.340	0.399	0.241	-0.301
motor	1.88	0.319	0.523	0.165	-0.353
nonplanning	2.36	0.428	0.569	0.0703	-0.449
BIS/BAS					
bis	2.33	0.499	0.413	0.924	0.0251
reward	2.80	0.542	0.690	0.831	-0.0560
drive	2.37	0.781	0.750	0.0910	-0.423
fun seeking	3.00	0.782	0.726	0.371	-0.232
Game experience					
competence	1.76	0.944	0.875	0.123	0.470
immersion	1.44	0.755	0.783	0.816	0.0755
flow	2. 45	0.873	0.790	0.501	0.216
tension	1. 20	1.10	0.913	0.303	-0.325
challenge	1. 54	0.697	0.613	0.972	0.0113
negative affect	0. 792	0.429	0.433	0.210	-0.390
positive affect	2.77	0.640	0.902	0.0558	0.565
Social presence					
empathy	1.57	0.717	0.803	0.715	0.118
involvement	1.66	0.937	0.752	0.540	-0.197
negative feelings	2.53	0.762	0.828	0.590	0.173
Post-game					
positive experience	1. 40	0.695	0.653	0.129	0.464
negative experience	0.347	0.315	0.499	0.402	-0.267
tiredness	0.958	0.652	0.701	0.256	-0.356
returning to reality	0.537	0.582	0.614	0.579	-0.179

**Fig 7:**
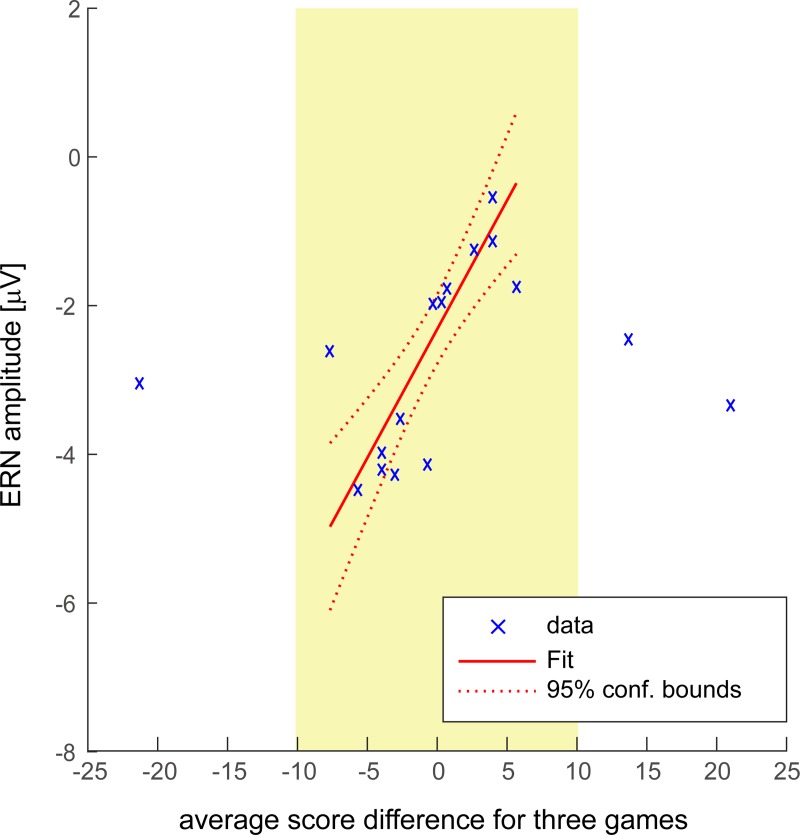
Correlation between ERN amplitude and the average score difference for the three games. Data in the yellow rectangle were used for fitting. ERN might be linearly modulated depending on who is winning the game and the relative score difference.

We also investigated the correlations between ERN amplitude and average final score difference for all three games. The results are shown in Fig 7 and S3 Table. When the score difference was within ±10, the negative potential of the ERN decreased when players were winning and increased when they were losing. Because the ERN changed linearly according to score differences within ±10, we fitted the ERN and scoring difference within a range of 10 points using weighted linear regression. We used the inverse of the variance of the score difference for all three games as the weight parameter. The average score differences and standard deviations for all three games are shown in Table 3. The results showed a significant correlation between smaller ERN amplitude and higher score difference (p < 0.001). ERN might be linearly modulated depending on who is winning the game and the relative score difference.

**Table 3:** Average score difference and standard deviations of for all three games for all participants.

Participants	Average score difference	SD
No. 1	-0.3333	1.53
No. 2	5.6667	3.51
No. 3	-4.0000	3.61
No. 4	13.7	6.66
No. 5	-0.667	1.53
No. 6	4.00	1.00
No. 7	2.67	1.53
No. 8	-21.3	5.51
No. 9	0.333	-1.53
No. 10	-5.67	-3.51
No. 11	21.0	-15.9
No. 12	-3.00	-4.00
No. 13	4.00	-3.61
No. 14	-7.67	-2.31
No. 15	0.667	-1.53
No. 16	-4.00	-1.00
No. 17	-2.67	-1.53

## Discussion

This study used a competitive-type consumer baseball game as an experiment task and measured brain EEG activity while participants played the game in conditions approximating those in normal daily life. We focused on two ERPs, the ERNs for batting errors and the P300 for attentional processing. A subjective evaluation of the game was obtained with the GEQ questionnaire and personality factors such as behavioral inhibition and activation were obtained with BIS11 and BIS/BAS scales. We investigated the relationship between these subjective evaluations and the ERPs for strike/ball events.

Strong ERN was observed in the fronto-central electrodes of the batter during the 200 ms before swinging strikes, but not in those of the pitcher. Thus, ERN amplitude differed significantly between batting and pitching conditions, and was not due to the influence of physical stimulus properties. Rather, it reflected the neural processing of errors that are experienced by missing the ball. The interesting finding is that the ERN was already observed about 200 ms before the error event onset. Therefore, we hypothesize that this ERN reflects the predicted error of one’s own batting, even when the miss has not yet occurred. This hypothesis is supported by another study in which ERN was reported to reflect error-prediction processes [33]. Our finding clearly showed that ERN is elicited by not only by actual errors but also by predicted errors represented in our minds.

P300 was mainly observed in the fronto-central electrodes of both batters and pitchers about 300 ms after swinging strikes. Because we did not find a significant difference in P300 between conditions, we can say that P300 does not represent error-specific brain activity. In this baseball game, the swing is the event that attracts the most attention from for both players. Therefore, we presume that P300 brain activity represents selective attention to salient and important events.

ERN was the most prominent ERP component in this study. Previous studies have argued that ERN reflects mismatch processing between response selection and execution [7]. Studies have also reported that the error detection system reflects cognitive and emotional responses to the deviation from predicted results [8,34]. In this study, ERN was observed about 200 ms before the error event onset. At that time, the ball was flying to the batter and had not yet reached the catcher’s mitt. For batting behaviors in a baseball game, the batter must predict the future location of the ball thrown by the pitcher. However, if the actual pitch’s course is different from the predicted ball course, neural processing related to misjudging the path of the ball might appear as ERN. However, because the pitcher cannot predict the batter’s tactics, the pitcher will not recognize his own mistake before the event onset. Therefore, we did not find larger ERN amplitudes before the event onset in video game pitchers. We assume that ERN is not observed in pitchers in real baseball games.

We observed a positive potential around 300 ms after the event onset. Although we interpreted this component as P300, it might actually be related to error positivity (Pe). Pe is a positive potential associated with errors that occurs after ERN [7,35]. Pe reflects additional processing after errors and is fundamentally different from error detection or response checking, which is reflected in the ERN [36]. Pe is similar to P300 in that it has the same polarity, is maximal at central-parietal sites, and peaks around 300 ms after error onset. Thus, Pe is often interpreted as a second P300. However, some studies suggest that Pe reflects a delayed stimulus-evoked P300 complex, which contributes to a seemingly response-locked ERP (see [37]). Despite this debate regarding Pe and P300, the positive potential around 300 ms after event onset in this study should be considered as P300. This is because the peak was observed in both players after swinging strikes. If this had been Pe, the amplitude would have been higher in batters than in pitchers. However, because these positive potentials did not differ, we interpret this potential as P300 for the cognitive processes such as attention to the judgement of the pitch. Note that Pe might have been buried inside the P300, but hidden because its influence on error processing was small. However, complete separation of Pe and P300 is difficult in this study and further research will be necessary to clarify the influence of Pe.

In this study, the ERN was evoked in the swinging strike events. However, we also confirmed that for the batters, called strikes elicited larger negative potentials than balls. Called strikes are classified as omission errors as the batter did not swing the bat when they should have. Although several clinical studies have reported behavioral results related to omission errors, very few studies have focused on EEG activity in these situations [38]. Although we did not observe a significant difference in ERN for called strikes between the batters and pitchers, we might have obtained omission-related ERN because the competitive baseball game increased error rates as well as participant’s intrinsic motivation, which might result in higher sensitivity to errors.

We also performed correlation analyses between ERN amplitude and self-reported evaluation scores obtained from BIS11, BIS/BAS, and the GEQ. We did not observe any significant correlation between ERN and BIS11 scores or any BIS/BAS item. These results suggest that the error-related potentials were not modulated by personality factors such as impulsivity or that variation in these traits was small across participants. The relationship between impulsive behaviors and ERN modulation is a topic currently under debate [39,40]. Past studies report that impulsive behavior does not necessarily lead to ERN modulation [41–44]. Concluding that impulsivity is directly involved in the modulation of ERN potentials is therefore difficult. For the GEQ items that evaluated the game experience, we found a trend for smaller ERN to be associated with larger scores for positive affect (*p* = 0.0558, *r* = 0.565). These findings suggest that ERN is suppressed when participants feel positive about the game, possibly because their optimism affects their perception of errors. This argument is also supported by the other results. Correlation tendencies were not found in items that represent negative feelings about the game (*p* > 0.1). It appears that items based on positive expression are predisposed to extract participant evaluations of the game.

There was a significant correlation between ERN and score differences in the range of ±10.When the score difference was within ±10, the negative potential of the ERN was suppressed when players were winning, but increased when they were losing. When players were losing, they cared more about making batting mistakes, and their neural response to swinging strikes was higher. In contrast, if players were winning, they should be less concerned. Therefore, even after a swing and miss, the game situation (i.e., winning) might not change, and the batter did not feel that the error was very serious. However, when players were losing or winning by a large margin (for example, 10 points), there was a different relationship between the score difference and ERN. It may be too early to draw the conclusion that the ERNs obtained with score differences of 10 points or more were changed linearly. When players were losing by a large margin, they likely lost motivation for the game. Even after a mistake, they might not have felt disappointed, which led to a weaker ERN. Conversely, when players were winning by a large margin, they might tend to underestimate batting difficulty and expect to hit with a high probability. If they swung and missed in this case, the deviation between predicted outcome and result would be large because they expected to hit the ball. Thus, swinging strikes might elicit a higher ERN magnitude. This hypothesis is supported by previous studies [33,45] in which the correlation between task performance and ERN were investigated. These studies reported that higher ERN amplitude was observed when the success rate was high. Conversely, when the success rate remained low, the ERN became relatively small. Thus, ERN may be modulated by intrinsic motivation, which is adaptively modulated by gaming situations.

This study has some limitations. First, we did not observe any significant correlations between ERN and personal factors. However, we found a significant correlation between the average score difference for three games and the ERN. In the baseball game, the win-lose situation for the player changes depending on the game score. The ERN might have changed according to the win/lose situation, rather than personal factors. Thus, the present study does not lead to the conclusion that personal factors do not change the magnitude of ERNs. Second, although we suggested that ERNs changed linearly according to the score difference, we assumed that ERNs obtained with score differences of 10 points or more changed nonlinearly. However, we did not observe a sufficient number of ERN data to validate this hypothesis. Third, most of the participants were male. Although the relationship between gender differences and ERN modulation is currently under debate, previous studies reported that ERN was affected by sex differences [46–48]. Therefore, we are unable to discuss the effects of sex differences on the current results. Fourth, we did not take the current base-path situation into consideration. For example, when batters commit swinging strikes, the feeling when they have runners in scoring position with two outs, is quite different from that when they have no runners on base with no outs. Future investigation is necessary to examine the relationship between the context of batting errors and ERN.

## Conclusions

We showed that ERN and P300 were evoked by ball/strike errors in a competitive consumer baseball game. P300 was evoked for swinging strikes, with no significant differences between batters and pitchers. We interpret this to mean that P300 reflected cognitive processes such as attention to the pitch outcome. In contrast, ERN for batters differed depending on the score difference. Because ERN amplitude was modulated by the relative score of the game, it can be a good metric for evaluating intrinsic motivation to play a game. We can provide entertainment content that matches the degree of difficulty appropriate for the user. If we can use ERN modulation to estimate the progress of a player’s skill and his/her degree of boredom, we can develop better learning and training methods for various types of games. The present experimental setting using a consumer game was able to increase intrinsic motivation to play the game and has helped us learn more about ERN modulation.

## Supporting information

S1 Table ERN and P300 amplitude data. (XLSX)

S2 Table ERN amplitude and self-reporting questionnaire data. (XLSX)

S3 Table ERN amplitude and score difference data. (XLSX)
